# P-1500. Evaluation of Imipenem-cilastatin-relebactam for the Treatment of Infections Caused by *Pseudomonas aeruginosa*

**DOI:** 10.1093/ofid/ofae631.1669

**Published:** 2025-01-29

**Authors:** Kaylee E Caniff, Xhilda Xhemali, Nikki Tran, Taryn A Eubank, Kevin W Garey, Yi Guo, Mei H Chang, Katie Barber, Mark Biagi, Travis J Carlson, Jeremy J Frens, Tamara Krekel, Venugopalan Veena, Wesley D Kufel, Amy L Carr, Jillian Hayes, James Sanders, Elisabeth Chandler, Rosanna Li, Julie Ann Justo, Kayla Antosz, Russell J Benefield, William Justin Moore, Jennifer K Ross, Athena L V Hobbs, Nicholas Mercuro, Brian Raux, Kristen Zeitler, Michael J Rybak

**Affiliations:** Anti-Infective Research Lab, Eugene Applebaum College of Pharmacy and Health Sciences, Wayne State University, Royal Oak, Michigan; University of Kentucky HealthCare, Farmington Hills, Michigan; The Ohio State Wexner Medical Center, Columbus, Ohio; University of Houston College of Pharmacy, Houston, Texas; University of Houston, Houston, TX; Montefiore Medical Center, Bronx, New York; Montefiore Medical Center, Bronx, New York; University of Mississippi, Jackson, MS; Swedish American Hospital, Rockford, Illinois; The University of Texas at Austin College of Pharmacy, San Antonio, Texas; Cone Health, GREENSBORO, North Carolina; Barnes-Jewish Hospital, St. Louis, MO; University of Florida, College of Pharmacy, Gainesville, Florida; Binghamton University School of Pharmacy Sciences, Binghamton, NY; AdventHealth Orlando, Orlando, Florida; AdventHealth Orlando, Orlando, Florida; UT Southwestern Medical Center, Dallas, Texas; Gulf Coast Medical Center, Fort Myers, Florida; Maimonides Medical Center, New York, New York; Dartmouth Hitchcock Medical Center, East Thetford, Vermont; University of South Carolina College of Pharmacy, Columbia, South Carolina; University of Utah Health, Salt Lake City, Utah; Northwestern Medicine, Chicago, Illinois; M Health Fairview University of Minnesota Medical Center, Minneapolis, Minnesota; Cardinal Health, Dublin, Ohio; Beth Israel Deaconess Medical Center, Boston, Massachusetts; Medical University of South Carolina, Charleston, South Carolina; Tampa General Hospital, Tampa, Florida; Eugene Applebaum College of Pharmacy and Health Sciences, Detroit, Michigan

## Abstract

**Background:**

The escalating prevalence of multidrug-resistant (MDR) *Pseudomonas aeruginosa* presents a serious threat to patient care due to its limited treatment options. However, imipenem-cilastatin-relebactam (IMI/REL) is a promising beta-lactam/beta-lactamase inhibitor combination with expanded activity against MDR *P. aeruginosa.* This study aimed to explore the patient characteristics, efficacy and safety of IMI/REL for treatment of infections due to *P. aeruginosa*.

**Table 1.**
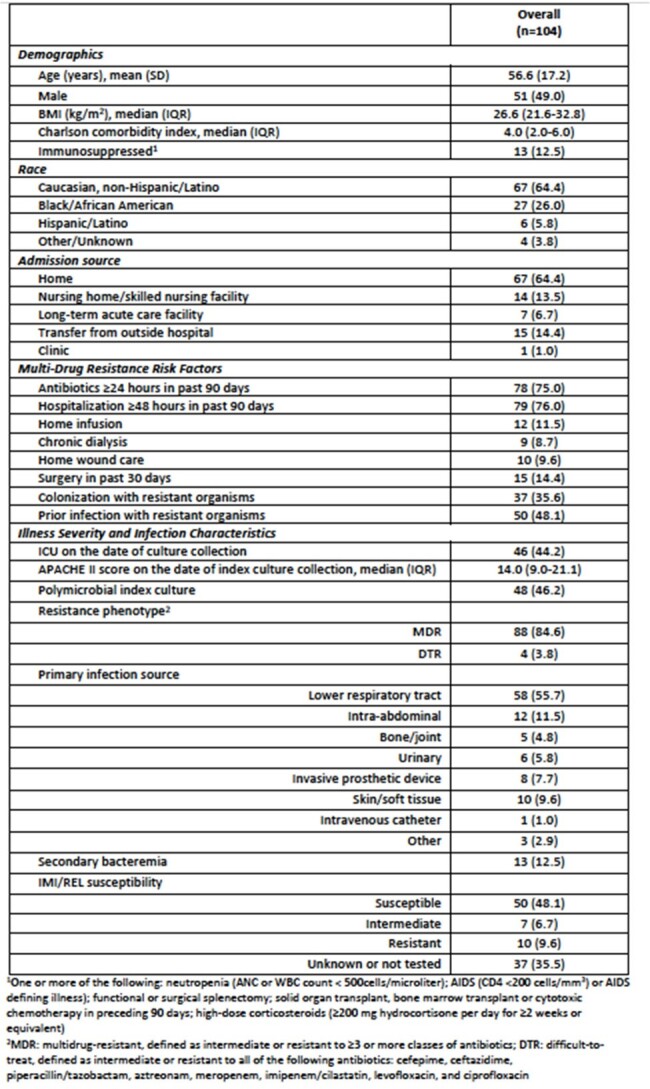
Patient and infection characteristics.

**Methods:**

This was a retrospective, observational, multicenter cohort study that included patients ≥ 18 years old who received IMI/REL for ≥ 48 hours for the treatment of an infection due to *P. aeruginosa.* The primary outcome was clinical success, defined as the resolution of or improvement in infectious signs and symptoms following initiation of IMI/REL until the end of therapy. Secondary outcomes included 30-day all-cause mortality, 30-day microbiologic recurrence, 30-day symptomatic recurrence and incidence of adverse drug reactions (ADRs).

**Table 2.**
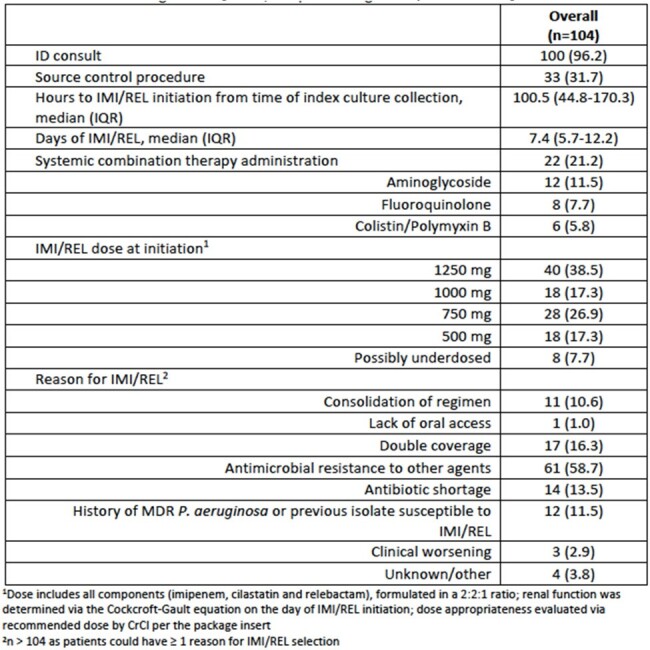
Infection management and IMI/REL prescribing.

**Results:**

There were 104 patients from nine U.S. medical centers included. The mean (standard deviation) age was 56.6 (17.2) years and the majority of patients were non-Hispanic Caucasian (64.4%). IMI/REL was predominantly used to treat lower-respiratory tract infections (55.7%) and 12.5% of cases developed secondary bacteremia. MDR *P. aeruginosa* was isolated in most patients (84.6%), while four patients (3.8%) were infected with a difficult-to-treat isolate. IMI/REL was initiated at a median (interquartile range) of 100.5 (44.8-170.3) hours from the time of index culture collection and was primarily selected due to the presence of resistance to alternative agents. Clinical success occurred in 73.1% of patients and 16.3% experienced 30-day all-cause mortality. Thirty-day microbiologic recurrence occurred in 14.4% of patients. ADRs occurred in six patients resulting in IMI/REL discontinuation in two cases.

**Table 3.**
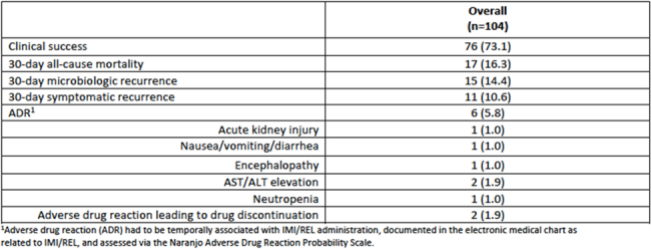
Clinical outcomes.

**Conclusion:**

To our knowledge, this is the largest report to date describing the use of IMI/REL for infections due to *P. aeruginosa*. The promising results herein justify the need for larger and comparative studies investigating the use of IMI/REL in this setting.

**Disclosures:**

**Kaylee E. Caniff, PharmD, BCIDP**, T2Biosystems: Honoraria **Kevin W. Garey, PharmD, MS**, Acurx: Grant/Research Support **Travis J. Carlson, PharmD, BCIDP**, Aimmune Therapeutics, Inc.: Speakers Bureau **Tamara Krekel, PharmD, BCPS, BCIDP**, Merck Inc: Honoraria **Wesley D. Kufel, Pharm.D., BCPS, BCIDP**, Merck & Co.: Grant/Research Support|Shionogi, Inc: Grant/Research Support **Amy L. Carr, PharmD, BCIDP**, Entasis: Advisor/Consultant|Ferring: Advisor/Consultant|Gilead: Advisor/Consultant|InflaRx: Advisor/Consultant|LaJolla: Advisor/Consultant|Melinta: Advisor/Consultant|MicroGenDx: Advisor/Consultant|Shionogi: Grant/Research Support **Jillian Hayes, PharmD, BCIDP**, GlaxoSmithKline: employee **James Sanders, PhD, PharmD**, Merck: Grant/Research Support|Shionogi: Grant/Research Support **Julie Ann Justo, PharmD, MS, FIDSA, BCPS**, Shionogi: Advisor/Consultant **Russell J. Benefield, PharmD, BCPS-AQ ID**, Paratek Pharmaceuticals: Grant/Research Support **Michael J. Rybak, PharmD, PhD, MPH**, Abbvie, Melinta, Sionogi, Merck, T2Biosystems: Advisor/Consultant|Abbvie, Melinta, Sionogi, Merck, T2Biosystems: Grant/Research Support|Abbvie, Melinta, Sionogi, Merck, T2Biosystems: Speaker

